# The Transmission of Diphtheria

**Published:** 1904-05

**Authors:** 


### SELECTIONS AND ABSTRACTS.

### THE TRANSMISSION OF DIPHTHERIA.*

This disease has prevailed at times in various parts of the world from a remote period, and has been described by writers in different countries since the time of Galen. In 1789 the disease was described by Bard of New York as *angina suffocativa,* and in 1828 Bretonneau of France, gave a clear and lucid description of its characteristics. Its contagiousness or communicability has for all time been recognized, but the immediate infective agent, the bacillus diphtheria, was discovered by Klebs in 1883, and afterwards studied and cultivated by Loeffler in 1884. Since then there has been much time and labor devoted to the study of diphtheria, and there is no doubt that the Klebs-Loeffler bacillus is the specific cause of true diphtheria. The germs are disseminated by the infected persons spitting, coughing, sneezing and picking the nose with the fingers, etc. Handkerchiefs, napkins, towels, bed-clothing, wearing apparel of any kind; cups, spoons or other dishes; pencils, books or other material used by the infected, are common vehicles for the bacilli.

Dr. W. H. Park reports that the bacilli have been observed to remain virulent in bits of dry membrane by Loeffler for fourteen weeks, by himself for seventeen weeks, and by Roux and Yersin for twenty weeks. Dried on silk threads, Abel reports that they may sometimes live one hundred and seventy-two days, and upon a child's plaything which had been kept in a dark place they lived for five months.

This disease is most often communicated by personal contact. The nurse or attendant, the members of the immediate family, those occupying the same house, in crowded and ill-ventilated institutions, or in close contact or association are most apt to contract the disease.

---

*Read by L. M. Powers, M.D., Health Officer of the City of Los Angeles, before the Los Angeles County Medical Association, February, 1904.

Every well-regulated community or municipality appreciating the fact that the isolation of the sick, and the disinfection of the dwelling and its contents, will limit the spread of disease, creates laws and makes rules for the isolation of persons sick with diphtheria. These regulations do not provide complete quarantine, inasmuch as egress and ingress is allowed certain members of the household, and those who have been exposed are not isolated, hence permitting the possibly infected to communicate with the uninfected.

The committee of the Massachusetts Association of Boards of Health concluded as follows:

First—It is impracticable to isolate well persons infected with diphtheria bacilli if such persons have not, so far as known, been recently exposed to the disease.

Second—It is not advisable as a matter of routine to isolate from the public *all* the well persons in infected families, schools and institutions, cultures from which show the presence of the diphtheria bacillus.

The committee believes that the danger from an infected healthy person depends on the age, habits, surroundings, occupation and intelligence of the individual, and all of these matters should be taken into consideration by the health authorities.

Dr. Westbrook modifies his assent to conclusion second as follows: "It may sometimes be impracticable to isolate from the public all the well persons in infected families, schools and institutions, though it should be done as a routine if at all possible."

It is impossible to maintain a complete isolation without a guard about the premises, for there are some who do not know enough to fear the disease or care to take precautions against transmitting it to others. Many persons thus exposed do not show clinical symptoms, and these are not isolated, but allowed to grow and disseminate the Klebs-Loeffler bacilli, and these bacilli may be non-virulent to some persons and virulent to others, as they enter the immune or non-immune.

The bacilli may remain in the throat or nose of a person for weeks, and at times are more or less virulent, or the system may be able to manufacture antitoxin for awhile and then from some cause of depression of vitality fail to supply sufficient antitoxin to resist the invasion of the germs, thus giving us sub-acute cases.

This disease may be mild, as we see in other contagious diseases, such as walking typhoid and smallpox cases, or even smallpox without eruption, so we have cases of diphtheria without membrane which may transmit the most fatal form to some other persons.

Many bacteriologists have studied the morphological properties of the diphtheria bacilli, with the view of ascertaining the virulent or non-virulent characteristics, but so far as I can learn, the only reliable means of determining the virulence of the germ is by guinea-pig tests, and this test would not be practicable for quarantine purposes.

The rules of the boards of health in all large cities to-day require cultures to be made showing the presence of diphtheria germs in the patient before placarding the house; also the absence of the germs in the throat and nose before releasing the patient and disinfecting the house, etc. This city and many others require but one culture to show the absence of germs in a patient, and rely upon the family physician to take the culture to release the patient, which, I am sorry to say, is oftentimes not as carefully taken to protect the public as it is to remove the quarantine.

It is of interest to note a summary of a report made by Dr. W. A. Macy, of an epidemic of diphtheria at the Willard State Hospital, New York, in which he says:

"Early in 1897 diphtheria was brought into the institution through employees who had been in contact with a case near by. There followed an epidemic, a moderate number of cases developing from time to time for six months, when it ceased without recurrence then or during the following year. In June, 1899, diphtheria broke out again, probably likewise imported, spread rapidly, seventy-five cases recurring, of mild type. It has continued, with intermissions of a month or two, till now, there being at present no actual diphtheria, but a number of germ cases in isolation.

"The condition is of a long-lasting epidemic, in an institution, mainly adults, of 2,700 population, under resident medical supervision, with a well-appointed laboratory used on so extensive a scale that every member of the community had cultures taken over and over again, all of which are recorded. It was observed:

"(1) That the type of the disease varies in different epidemics; in the first, 1898, nearly every one exposed, even slightly, took diphtheria, but the course of the disease was mild; in the present epidemic it was much less infectious, but much more virulent in character.

"(2) The germs of diphtheria can maintain activity with much persistence outside the body and resist the action of ordinary disinfectants. Recurrence of the epidemic after all the people were found germ free was found to occur in rooms which, after occupation by the sick, had been subjected freely to formaldehyde, sulphur fumigation, washing with bichloride, hot soapsuds or soda solution, both walls and woodwork. Twenty-five cultures were often taken before a building was found free.

"(3) It was found that cases discharged from quarantine after three negative cultures showed Klebs-Loeffler germs present; the sick were discharged only after three negative cultures, taken on alternative days. None of these thus tested were found afterwards to show the disease germs, but nothing short of this was found trustworthy.

"(4) Presence of the germs in healthy throat was found; they persisted there for three weeks; cultures were almost or quite as pure as from membranous cases; virulence tests in the laboratory showed them to be as dangerous as those taken from clinical cases. Membranous diphtheria followed exposure to such, and even at second-hand, for in one clear instance a person associating with a germ case without sore throat carried in his clothing the disease in the virulent form to a family living several miles distant, bearing germs taken from that case. It would appear, then, that bacteriological diphtheria is as potently dangerous as clinical.

"(5) Change in the shape of germs attended convalescence; when these 'degenerate' forms occurred, the disease generally soon yielded. There was reason to think that these attenuated forms may, under conditions favorable to rapid development, increase in strength, but that they have not sufficient virulence to be actually dangerous.

While in membrane cases the bacilli followed a definite life history, those from a normal throat may differ daily, a fact important to bear in mind."

In June, 1903, there were a number of diphtheria cases reported to the health officer from the neighborhood of Westlake Park, and upon inspection I learned that many familes where the disease existed were supplied with milk from the Westlake Dairy. I made cultures from the utensils, and the throats of two children who helped about the dairy and delivered milk to customers. All of these cultures proving negative, the proprietor of the dairy was instructed to construct a suitable milk-house, to clean up the corral, and to boil or sterilize the milk-bottles before filling them. We had observed that the bottles were only washed in warm water and and soda before being used. As I could not find any evidence of diphtheria at the dairy, I concluded that the disease was being spread by refilling unsterilized bottles which had been left in houses where the disease existed.

The disease ceased to spread and disappeared until October seventh, when six cases were reported from the same district and in families supplied from the same dairy. October eighth five cases were reported, etc., from the same district; and October ninth four cases were reported, etc. I visited the dairy, and, finding the proprietor and family absent, and no one there to answer any questions except the milker, who could not speak English, I made cultures of the brush used to clean the bottles, the water from the tub the bottles had been washed in, and of an uncleaned milk can. These cultures showed negative. October tenth five cases were reported, etc. October eleventh four cases were reported, etc.; and again I visited the dairy and found the family (consisting of the dairyman, his wife and two children, and a milker). I made cultures from the throats of all five persons. The throats of the wife, the milker and one daughter appeared about normal. The elder daughter had enlarged tonsils, with catarrhal pharyngitis. The proprietor's left tonsil was enlarged and inflamed. I again made cultures from the milk utensils, and the report from Dr. Leonard, the bacteriologist, was as follows: "Cultures from the two girls positive; the proprietor, wife, and the milker, negative. The cultures from the milk utensils negative, with regularly stained suspicious bacilli in the culture from the brush." October twelfth I again visited the dairy, placarded the house, and stopped the sale of milk. I made cultures from the throats of the proprietor, his wife, and the milker which were reported upon as positive in the cultures from the throats of the proprietor and the milker, but negative in that taken from the throat of the dairyman's wife. October thirteenth ten cases were reported from the same district in families which were supplied with milk from the same dairy.

During the period from October seventh to October seventeenth there were in all thirty-three cases of diphtheria reported in thirty families of one hundred and ten families that received milk from this dairy.

On October twenty-first there were four cases of diphtheria reported from Angeleno Heights and neighborhood. Upon investigation, I learned that the familes in which these cases existed were supplied with milk in bottles by the Angeleno Dairy. I visited the dairy and made cultures from the throat of one of the proprietors, the milker and two boys, who had occupied the same building with the absent proprietor and the milker at the dairy; also from the brush used for cleaning the bottles, and from the cloth through which the milk was strained into the large mixing-can.

On October twenty-second, there being other cases reported from the same district, I again visited the dairy and made cultures from the utensils and the throats of the same parties as the day before, but could not ascertain the whereabouts of one of the proprietors, who was also absent the previous day. It was stated that he had gone down town to consult a physician about a sore hand. I returned to the office, to learn that the man that I was looking for was in one of the hotels, and that a physician had left a culture from his throat in the office. I saw the young man, who had a well-marked clinical diphtheria. I returned to the dairy with the milk inspector and placarded the building which the sick proprietor and the milker had occupied, and stopped the sale of milk. October twenty-third the cultures from the throats of the two proprietors showed positive, and all other cultures negative, except the one from the straining-cloth, which showed regularly stained suspicious bacilli. October twenty-seventh a culture taken from the throat of the milker showed Klebs-Loeffler bacilli.

The throat of the milker and one of the drivers or proprietors showed no membrane.

There were in all thirty-five cases reported from thirty-three families receiving their milk from this dairy between October 21 and November 1. The two proprietors delivered the milk to the customers, and their route or district overlapped a portion of the district or route of the Westlake Dairy. I found that their bottles had not been sterilized by boiling or steaming. The bottles were delivered to families, there used as part of the household furnishing, returned to the dairy, where, in each dairy, the warm water and soda washing was considered sufficient. I also have reason to believe that the bottles were sometimes refilled by the first dairyman on the wagon without being returned to the dairy.

On inspecting each of these dairies, I closely scrutinized the udders of the cattle for diphtheritic sores or ulcers, and found nothing suspicious.

While placarding a house where the mother was the infected member of the family, I saw two young children in good, healthy condition, and on inquiry learned from the father that the two children were not permitted to drink milk until it had been cooked, but that the mother usually tasted of the milk to see if it was sweet before cooking it for the children. I think the milk became infected in both dairies from the use of bottles which had been delivered to and returned from houses where there were mild cases, or previous to the reporting and isolation of more severe cases. After the dairyman became infected, he, no doubt, infected the bottles by handling them before delivery to his customers. In connection with this, I desire to notice a report made by Dr. Denny in the journal of the Massachusetts Associated Boards of Health, April, 1900, which reads as follows:

"In October, 1899, two children in a milkman's family were taken sick with diphtheria and were sent to the hospital. Realizing that others in the same house who were handling the milk might have diphtheria bacilli in their throats, and that the milk might be so infected, Dr. Chase made cultures from all the well members of the milkman's household. All were negative at that time. About three weeks later a number of cases of diphtheria began to appear among the milkman's customers. Cultures were again made from the well members of the household, and three healthy men were found to have the bacilli. Two of these men did the milking. The bacilli in their throats were very abundant, and were shown to be virulent by the inoculation of guinea-pigs. In four families of the milkman's eight Brookline customers, there were twelve cases of diphtheria; at least six cases occurring among his customers outside of Brookline. Dr. Chase has shown very clearly that these eighteen persons were infected through the milk by the bacilli from the two healthy milkers. A fourth healthy man with the bacilli in his throat was found by Dr. Chase among thirty-one adults who were known to have been exposed by drinking the infected milk."

The transmission of diphtheria by the lower animals has been reported many times, but in many cases not substantiated. Dr. Moore, in "The Pathology of Infectious Diseases of Animals," states that "A comparison of the bacillus of diphtheria in man (Klebs-Loeffler) with those described from diphtheria in fowls, shows that, morphologically and in their pathogenesis for experimental animals, the organisms are in no way alike. There is also a marked difference in the nature of the exudate in fowls and in man. The non-identity of these diseases has been clearly pointed out by Menard. Although these maladies are shown by several observations to be unlike in their etiology and the character of their lesions, the transmission of fowl diphtheria to the human species, and vice versa, is affirmed by several writers."

I have seen well-marked cases in cats, and Dr. Eddy, of this city, sent a culture from the throat of a cat which showed the barred forms of the germ. This cat belonged to a family in which diphtheria existed.

It is a well-known fact that the construction of sewers and the abandonment of cesspools and privy vaults is generally accompanied by an increase of diphtheria in the immediate section, which is, no doubt, caused by the infected contents of the cesspools and privy vaults being brought to the surface by the filling and the feet of those working in or about the same, and the bacilli conveyed to the carpets in the homes by the shoes of the workmen, or, after the infected contents of cesspools or vaults being dried, conveyed by the wind into the houses, or, perhaps, directly into the throats of the children. We know that often the refuse from the sick-room, such as old rags, paper and the contents of cuspidors, are emptied into cesspools without being disinfected, thereby infecting the cesspool.

McFarland reports that the bacilli in sand, exposed to a dry atmosphere, die in five days in the light, and in sixteen to eighteen days in the dark; where the sand is exposed to a moist atmosphere, the duration of vitality is doubled. In fine earth they remain alive from seventy-five to one hundred and five days in dry air, and one hundred and twenty-five days in moist air. In the city of Los Angeles we have observed that following a wind-storm there is always an increase of diphtheria. While it may be possible that the particles of dust, etc., in the air during wind-storms irritate the throats of already infected persons, and thereby develop the disease, yet we have all seen cases of primary laryngeal and bronchial diphtheria without any clinical or bacteriological evidence in the nose or pharynx; therefore, we conclude that the air must have conveyed the germs to the point of infection.—*Southern California Practitioner.*

